# The contribution of high-resolution GC separations in plastic recycling research

**DOI:** 10.1007/s00216-023-04519-8

**Published:** 2023-01-18

**Authors:** Delphine Zanella, Monica Romagnoli, Sofia Malcangi, Marco Beccaria, Tatiana Chenet, Chiara De Luca, Fabio Testoni, Luisa Pasti, Ugo Visentini, Giampiero Morini, Alberto Cavazzini, Flavio A. Franchina

**Affiliations:** 1 Giulio Natta Research Center, LyondellBasell Italy, Piazzale Donegani 12, 44122 Ferrara, Italy; 2 Department of Chemical, Pharmaceutical, and Agricultural Sciences, University of Ferrara, Via L. Borsari 46, 44121 Ferrara, Italy; 3 Department of Environmental and Prevention Sciences, University of Ferrara, Via L. Borsari 46, 44121 Ferrara, Italy

**Keywords:** Gas chromatography, Comprehensive two-dimensional gas chromatography, Mass spectrometry, Pyrolysis oil, Hydrocarbons, Plastic waste composition, Molecular characterization

## Abstract

One convenient strategy to reduce environmental impact and pollution involves the reuse and revalorization of waste produced by modern society. Nowadays, global plastic production has reached 367 million tons per year and because of their durable nature, their recycling is fundamental for the achievement of the circular economy objective. In closing the loop of plastics, advanced recycling, i.e., the breakdown of plastics into their building blocks and their transformation into valuable secondary raw materials, is a promising management option for post-consumer plastic waste. The most valuable product from advanced recycling is a fluid hydrocarbon stream (or pyrolysis oil) which represents the feedstock for further refinement and processing into new plastics. In this context, gas chromatography is currently playing an important role since it is being used to study the pyrolysis oils, as well as any organic contaminants, and it can be considered a high-resolution separation technique, able to provide the molecular composition of such complex samples. This information significantly helps to tailor the pyrolysis process to produce high-quality feedstocks. In addition, the detection of contaminants (i.e., heteroatom-containing compounds) is crucial to avoid catalytic deterioration and to implement and design further purification processes. The current review highlights the importance of molecular characterization of waste stream products, and particularly the pyrolysis oils obtained from waste plastics. An overview of relevant applications published recently will be provided, and the potential of comprehensive two-dimensional gas chromatography, which represents the natural evolution of gas chromatography into a higher-resolution technique, will be underlined.

## Introduction

**Fig. 1:**
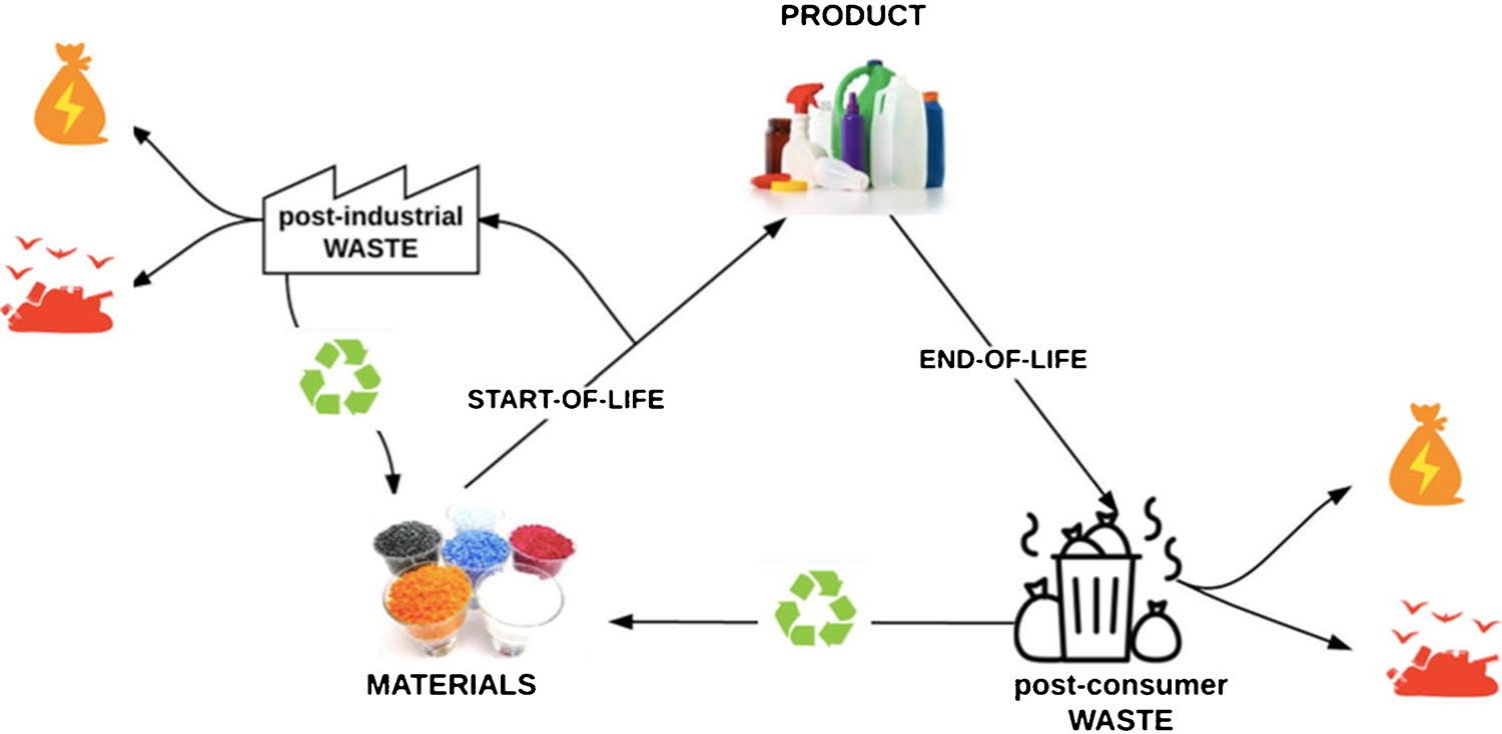
Lifecycle of polymer materials. Depending on the chosen route, polymers will end up in recycling, energy recovery, or landfill, with permission from [1]

Plastic waste originates mainly from industrial activities and consumer products, and it can be grouped into two categories: pre-consumer or post-industrial plastic wastes and post-consumer or municipal plastic wastes (MSW) (Fig. 1).

Post-industrial plastic waste is a solid waste generated during the manufacturing process, which never arrives to consumers, and includes rejected products, cuttings, trimmings, runners from injection molding, and residues from granulation. This is usually uncontaminated by organic matter or pollutants such as paper, wood, or other plastics, and it is often mono-material or multi-material of known composition (polymers and their respective amounts in the case of plastics). Post-industrial plastic waste usually remains in the company where it is produced or is handled business-to-business [1].

On the other hand, post-consumer plastic waste generated at the end-of-life of the product typically consists of mixed plastics of unknown composition. It is likely contaminated with organic fractions such as food waste or other non-polymeric materials (metal or paper). Post-consumer waste is handled by municipalities and is usually well-tracked throughout Europe [1].

In 2022, the global production of plastics reached 367 million tons among which 55 million tons were produced in Europe [2, 3]. Plastic waste fractions constitute a large portion of municipal solid waste, accounting for roughly 10–13% of the entire MSW throughout the world [4, 5]. The growing awareness of problematics associated with plastic waste has resulted in growing advocacy for the introduction of a circular economy model. Problems associated with plastic waste include the environmental impact of pollution caused by poor waste management practices, such as landfill, and the consumption of natural resources.

The establishment of the circular economy paradigm is the ideal solution to reduce the generation of waste and the consumption of natural resources by recycling and reusing plastics at the end of their life span. For the EU, “in a circular economy, the value of products and materials is maintained for as long as possible; waste and resource use are minimized, and resources are kept within the economy when a product has reached the end of its life, to be used again and again to create further value” [6].

The latest European report on the circular economy for plastics stated that, in 2020, of the 29.5 million tons of post-consumer plastic waste collected, 35% have been recycled [3]. Although it represents a recycling rate increase of 15% from 2018, more than 65% of post-consumer plastic waste was still landfilled. Therefore, to move towards this circular economy model, efficient recycling processes are highly needed and should be further optimized to produce high-quality feedstock.

**Fig. 2:**
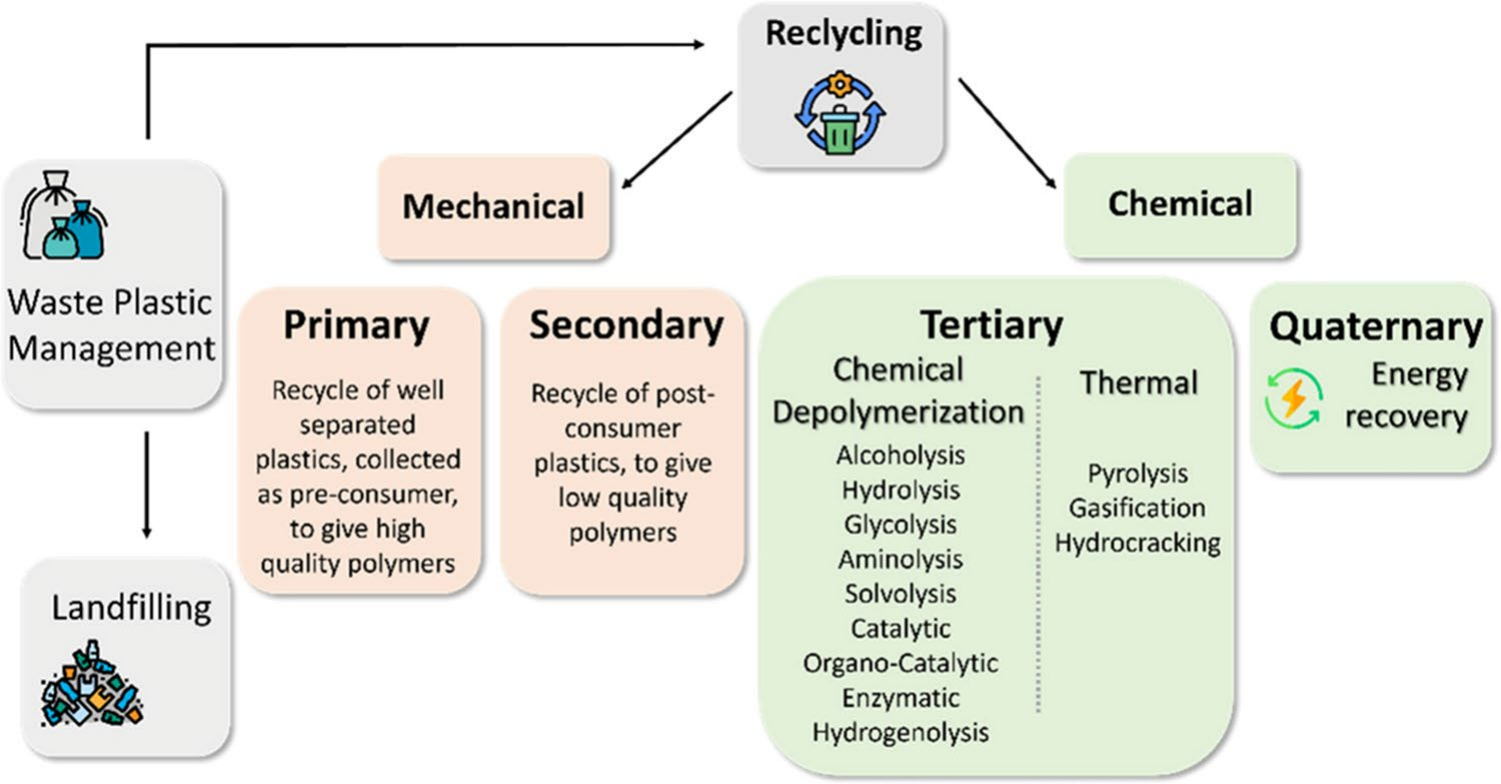
Overview of the plastic recycling techniques from [7]

Nowadays, various recycling technologies are present, as can be seen in Fig. 2, and four main categories can be identified: primary recycling, secondary recycling, tertiary recycling, and quaternary recycling (for energy production).

Primary and secondary recycling consists of mechanical processes enabling the recycling of single-polymer plastics such as polyethylene (PE), polypropylene (PP), and polyethylene terephthalate (PET). Primary recycling involves the direct reuse, ideally maintaining the initial polymer properties, of polymer scrap produced during manufacturing. Regarding secondary recycling, prior separation, purification, and preparation (shredding/melting) of plastic waste are required. Contaminated and/or complex plastic waste is highly challenging to recycle mechanically. In addition, this process is often associated with the loss of polymer properties due to the mechanical stress and photo-oxidation occurring during the process [8–10]. Post-industrial waste is usually homogenous and of a higher quality than post-consumer waste, which favors its mechanical recycling. When mechanical processes fail because of excessive contamination, separation difficulties, or excessive deterioration of polymer properties, tertiary or quaternary processes can be applied. The latter, or energy recovery, consists of the incineration of plastic wastes and the recovery of energy through the direct production of heat and/or electricity.

The advanced or chemical recycling (i.e., tertiary recycling) covers various techniques aiming at the decomposition of plastic waste materials into smaller molecules by means of chemical processes [11]. Plastics are indeed synthetic polymers consisting of long hydrocarbon chains that can be broken down by using pyrolysis processes. The degradation of long-chain polymers into their constituents (i.e., monomers, oligomers) makes these a suitable source of feedstock to produce new plastics and petrochemicals. Advanced recycling is considered a promising management option for post-consumer plastic waste, because of the mixed and often unknown composition and contaminated nature of this type of waste [9, 10]. Advanced recycling technologies have already been successfully applied to treat mixed plastic waste affected by impurities.

The main chemical recycling processes in use include hydrocracking, pyrolysis or thermal cracking, and catalytic cracking [8]. In the hydrocracking process, high hydrogen pressure and elevated temperature are used to convert larger hydrocarbon molecules into smaller ones, which is commonly used to upgrade heavier fractions of crude oil. The pyrolysis process requires intense heat and pressure in an inert atmosphere, and it produces a liquid pyrolysis oil (PO), gas, and solid char, which are highly valuable for industries. As implied by the name, catalytic cracking involves the use of a proper catalyst to conduct the depolymerization process, reducing the time and temperature of the cracking, and thus the energy consumption [12]. The produced PO is characterized by a narrower hydrocarbon distribution representing a lighter oil fraction compared to non-catalytic pyrolysis. Lighter oil fractions are highly desired since their properties are close to those of conventional fuels. More importantly, the process parameters (temperatures, residing times, catalysts, etc.) affect the composition and the distribution of the fractions. Additional information on chemical recycling processes can be found in the literature [8, 13].

Advanced or chemical recycling of plastics could significantly push the transition from a linear to a circular economy by extending the potential feedstock production, providing high production yield while minimizing waste. The fact that new feedstocks can be produced in rather simple processes via pyrolysis and catalytic cracking is very promising, from different viewpoints. Indeed, advanced recycling represents an area of intense research and development [14].

However, a series of unwanted chemicals deriving from the degradation mechanism of plastics or external contamination exist and are needed to be monitored. Not only they can affect the long-term properties of polymers (mechanical properties, aspect, etc.), but also, they can be released from the polymeric matrix, migrating into the environment or into diverse contact materials (food, water, etc.). Altogether, these effects alter the quality of the final plastics. In other words, we can say that the molecular composition of the plastics and derivatives (i.e., pyrolysis oils) contains information on the origin/type, quality, and production/process conditions used.

POs need to be further processed in steam crackers to produce light hydrocarbons. Strict specifications in terms of composition exist for feedstocks to be suitable for steam cracking [15]. Therefore, an in-depth chemical characterization of the produced PO is required. Several challenges are associated with this task due to the variability and complexity of plastic waste materials, and the different process conditions [15, 16]. Significant variations within the hydrocarbon composition (i.e., PIONA composition—paraffins (P), iso-paraffins (I), olefins (O), naphthenes (N), and aromatics (A)), up to 30 wt% can be expected [16]. Such variations can be explained by the composition of the plastic waste. Pyrolysis oils produced from PP-rich waste are known to contain high amounts of branched iso-olefins and diolefins whereas PE-rich waste yield high content of olefins and linear paraffins [15].

Since olefins highly contribute to coke formation (i.e., unwanted carbonaceous deposits) in steam cracking and that the heteroatom-containing species, e.g., oxygen, nitrogen, sulfur, and chloride, not only can cause environmental issues and/or compromise further processing of POs but also degrade the catalyst if not properly accounted for, a careful and exhaustive characterization of the oil is required [17, 18] to adjust pyrolysis conditions.

The characterization of POs can be performed similarly to classical petrochemical hydrocarbon streams; however, their composition is significantly different [16]. Spectroscopic techniques, such as Fourier-transform infrared spectroscopy (FT-IR) and nuclear magnetic resonance (NMR), were proven useful to determine bulk properties, the functional group distribution, and the chemical nature of hydrogen and carbon atoms in oil [19, 20]. NMR analysis even enables the distinction of different types of hydrocarbon groups, such as aliphatic and aromatic. However, a detailed molecular characterization of POs cannot be obtained by using solely spectroscopic methods. Hyphenated techniques, based on the chromatographic separation of the complex pyrolysis mixtures in their constituent compounds, are of paramount importance in this field of research.

Gas chromatography (GC) separation is probably the most refined way to approach a detailed analysis of hydrocarbon mixtures. However, the complexity of this kind of samples represents still a big challenge and requires high selectivity and highly efficient separations. Even though additional pre-separation and purification steps can be adopted before the GC separation, sample preparation techniques will not be discussed in this review, but the interested reader is directed to the literature for more information [21, 22].

A clever approach to gain in selectivity is the exploitation of comprehensive multi-dimensional GC (GC×GC) [23, 24]. This technique combines and exploits the selectivity of two different stationary phases in a single analysis. The resulting 2D chromatograms are characterized by ordered elution regions, grouping the compounds belonging to the same chemical class (i.e., with similar physico-chemical properties). In addition, the gain in sensitivity, thanks to the band compression of the modulation, greatly supports the detection of heteroatom-containing compounds or those contaminants which are present at trace levels, and that can be difficult to determine using conventional GC methods.

Of course, to fully exploit the power of GC×GC for the characterization of such complex mixtures, a proper universal or selective detector must be considered [25].

In this regard, mass spectrometry (MS), without doubts, provides the most detailed molecular information of sample constituents. However, when multi-class quantitative analysis is sought, the use of universal detectors (e.g., the flame ionization detector, FID) may give more accurate results. That being said, an ultimate detector does not exists and, instead, a combination of them would be the most advantageous choice (for example, a parallel and complementary MS and FID information).

**Fig. 3:**
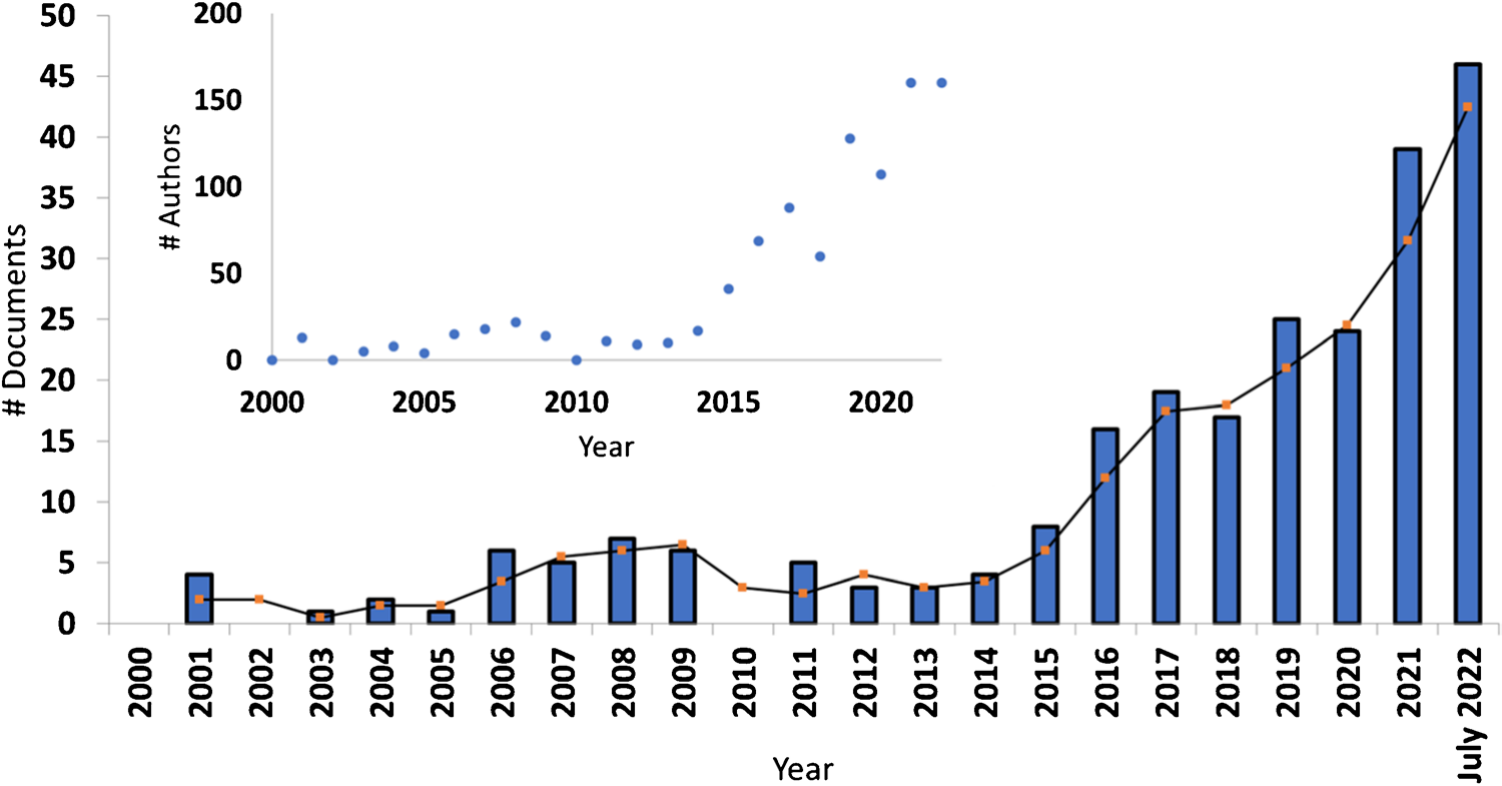
Temporal trends of research articles related to plastic pyrolysis research. The bar graph relates to the number of published papers and the continuous line is the moving average. The inset reports the number of publishing authors on the same temporal window. Keywords: plastic pyrolysis oil (and) GC (or) gas chromatography. Source: Scopus

The relevance of plastic management and reuse through pyrolysis is also supported in the active research on this topic. Although a slight increase in publications related to plastic pyrolysis is present in early 2000, a steep increase has been observed since 2015 (Fig. 3). The same trend can also be observed for the number of active authors (inset of Fig. 3), indicating that more researchers are actively participating in such a research topic.

This review is devoted to the high-resolution GC and GC×GC analysis of POs produced from waste materials, and more specifically from plastics. For other types of POs, for example, those originating from biomasses, the reader is directed to the literature [26, 27].

## GC separations in the context of plastics recycling and reuse

Various physical characterizations are routinely performed on bulk materials or their products, such as the density, cloud point, flash point, and color. In the plastics industry, GC separations are nowadays used routinary for quality assessment and chemical characterization of the final virgin polymers. The virgin plastic is a plastic material which is directly produced from a petrochemical feedstock and has not been subjected to an earlier use.

The use of GC becomes particularly relevant when it can be used to replace physical measurements. An example is the use of GC as an accurate alternative to distillation for measuring boiling-point ranges of hydrocarbon mixtures. In this type of analysis, known as simulated distillation, the separation of hydrocarbon components is performed on a non-polar column with an FID. Such analysis enables the determination of the relative yields of different hydrocarbon ranges, which represents valuable information for refining processes [28–30].

In a recent study, simulated distillation was used to determine the optimal cracking temperatures to maximize product yields (for tailored carbon ranges). Changes in the physical and chemical properties of pyrolysis oil were observed with respect to the pyrolysis temperature. It has been reported that at cracking temperatures of 450 °C the naphta- and kerosene-cuts were predominant, whereas 500 °C was the optimal temperature to produce light vacuum gas oil cut [31]. The determination of the true boiling point for mixed plastics PO is also valuable to enrich existing mathematical models able to predict the properties of POs.

Such an approach was also used to determine the impact of residence time on the yields and structure of the products [32]. The authors showed that the residence time had a significant impact on the yields of volatile products. In addition, the formation of aromatic compounds and increased degradation were observed with longer residence time. Other applications of GC separations regard the determination of specific components which can be regulated with maximum levels or can alter the quality of the final product. Important uses of GC also include the determination of trace levels of monomers, the monitoring of residual polymerization initiators and/or catalysts, the quantification of additives, and the evaluation of their degradation products [33]. Additional degradation products can also be evaluated together with odorous components [34].

The characterization of additives and by-products within the polymeric matrix is highly relevant to the recycling process. The transfer of additives into new recycled products requires particular attention since the additives can contain regulated substances, such as compounds classified as persistent organic pollutants or phthalates [33]. In addition, the recyclability of plastic materials can be directly impacted by additives: some additives can promote the degradation of plastics during the recycling process due to the formation of pro-oxidants, but they are also responsible for shortened plastic lifetime [33, 35].

To better address the challenges related to coelutions and sample complexity, GC coupled to MS (GC–MS) can be considered a reliable method. In a recent study, volatiles and semi-volatiles present in recycled low- and high-density PE have been characterized by GC–MS. The authors highlighted that among the 134 compounds identified, additives and additive degradation products represented 40% for/in the LDPE and 20% for/in the HDPE, which could restrain its reuse for high-added-value applications [36]. It has also been shown that phthalates, commonly used as plasticizers, could persist in plastics during the recycling process, therefore resulting in potential accumulation in recycled products [37]. GC–MS has also been successfully used to follow the evolution of selected additives and derived substances found in PP under solar stress [38].

Over the past years, the coupling of analytical pyrolysis to GC–MS (Py-GC/MS) has attracted growing attention to characterize additives from plastic materials [35]. One of the main advantages of such a technique is the minimal sample preparation required, thus reducing potential bias and contaminations. Indeed, most of the studies rely on solvent extraction methodologies to extract additives from plastic matrices, which introduces some degree of selectivity towards defined classes of analytes according to their physico-chemical properties.

Multi-shot Py-GC/MS enables the determination of additives and other organic compounds at the surface of the sample and the type of polymer in a single analysis using different temperatures. The first shot consists of the thermal desorption of organic compounds and additives without degrading the polymeric matrix, whereas the second shot, conducted at significantly higher temperatures, consists of the flash pyrolysis of the polymeric matrix giving information on its composition [35, 39, 40].

**Fig. 4:**
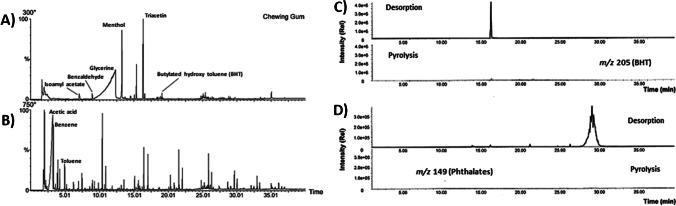
**A** Thermal desorption chromatogram at 300 °C; **B** Pyrogram at 750 °C of chewing gum; **C** Corresponding MS ion current at 205 m*/z*; **D** corresponding MS ion current at 149 *m/z*. Reproduced with permission from [41, 42]

To illustrate this concept, a chewing gum sample has been analyzed using multi-shot Py-GC/MS at 300 °C (first shot) and 750 °C (second shot) [41]. The chromatogram resulting from the thermal desorption at 300 °C of the chewing gum (Fig. 4A) resolved the organic additives present in the sample, such as the triacetin used as plasticizer and flavoring agents such as menthol, and isoamyl acetate. Conversely, the pyrogram at 750 °C (Fig. 4B) enabled the characterization of the polymeric nature of the gum, where the repeated oligomeric pattern, typical of the polyisobutylene, and the presence of acetic acid, benzene, and toluene characteristic of polyvinyl acetate, can be clearly observed.

It has also been demonstrated that the first thermal desorption step (conducted at 300 °C) was efficient for the complete removal of additives from the polymeric matrix [42]. As can be seen in Fig. 4C–D, phthalate plasticizers (*m/z* 149) and antioxidant butylated hydroxytoluene (BHT) were found in the thermal desorption chromatogram but not in the pyrogram.

Another valuable use of the GC separation concerns the determination of the composition of a mixture in substantial detail, which could be associated with the profiling or non-targeted analysis, which are typically used in other fields of application [43–46]. Such detailed analysis becomes particularly relevant in the more actual landscape of recycled plastics. As mentioned earlier, the plastic POs indeed are considered a potential new source of cracker feedstock; therefore, their in-depth molecular characterization is crucial, for example, to tune the cracking process.

Detailed hydrocarbon analysis (DHA) is an example of multi-targeted analysis requiring long high-resolution GC columns (100 m) and therefore long analysis run time (typically about 3 h per run) to determine the individual hydrocarbon components of fuels. Having such detailed information is important for modeling the operating margins [47]. DHA analysis was developed and implemented by the more-established fossil fuel research, from which most of the know-how and practices can be borrowed for the study of POs from plastics.

The DHA method not only provides information for the individual components but can also provide the quantification of the compound classes as weight percentages in the sample: it is worth noticing that the amount of information is dependent upon the chromatographic resolution of the separation system [48]. Indeed, the DHA method enables estimating the PIONA composition. As previously mentioned, strict specifications, established by the petrochemical industry, exist for the hydrocarbon composition of the feedstock. The total paraffin content, i.e., paraffins and iso-paraffins, should account for a minimum of 52 wt% of the materials, while olefins should be less than 0.8 wt% [49].

Nevertheless, coelutions and lack of comprehensive identification of analytes complicate these types of determinations and can affect the accuracy of the results. This is especially true for heavier hydrocarbon fractions, in which the compositional complexity increases proportionally. Consequently, one dimension GC method (1D GC) is often not sufficient to separate all components from one another, and high-resolution GC×GC methodologies are more appropriate.

**Fig. 5:**
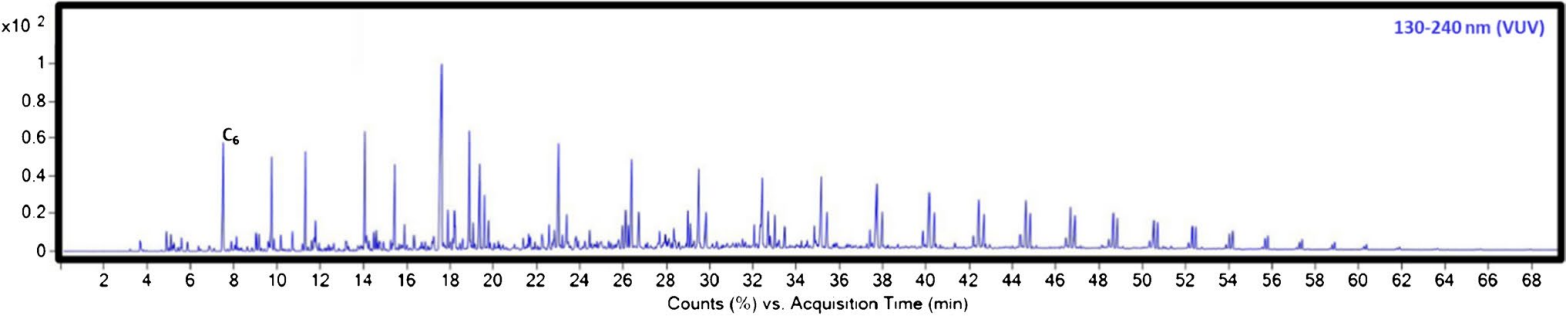
GC-VUV chromatograms (average 130–240 nm) of PO blend ranged from C_4_–C_32_. Reproduced with permission from [16]

Classically, DHA is carried out using the universal FID, thanks to its more uniform response factor among different chemical families and the greater linear dynamic range, compared to other detectors. Recently, the development of vacuum ultraviolet spectroscopy (VUV) detector technology has proved to be valuable for this kind of analysis, adding some additional selectivity and identification power with the selection of specific wavelengths [16]. Figure 5 shows the GC-VUV separation of a PO blend, in which the authors quantified the hydrocarbon classes according to PIONA from C_4_ through C_30_ (and higher). Details of the oil type/origin have unfortunately not been reported by the authors, but it is possible to speculate on the carbon number range and to observe the characteristic patterns. These sequences appear as consecutive peak triplets or quartets for the different carbon numbers, typical of polymer analysis. These peaks are originated by a random scission mechanism that leads to a fragmentation of the initial polymer chain into smaller straight alkanes, alkenes, and dienes, containing between 9 and 32 carbon atoms, depending on the polymer type and pyrolysis conditions [50, 51].

Even though feedstocks from fossil sources have been investigated more deeply, plastic-based sources have a vastly different hydrocarbon composition. PE or PP-based plastics will have a high olefin content whereas PS-based plastics will have a high aromatic content. In contrast, polyethylene terephthalate (PET) will have both a high olefin and aromatic content and may contain oxygenates as well. The current methodologies for evaluating the hydrocarbon composition of liquid hydrocarbon feedstocks are well-established for fossil-based materials [52].

However, they can turn out to be insufficient for determining in detail the hydrocarbon composition of mixed waste plastics POs. To fully characterize the hydrocarbon composition and potential contaminants of plastics POs, more accurate and high-resolution methodologies are better suited.

## GC×GC suitability and relevant applications

The universal benefit of high-resolution separations has undoubtedly advantages in unraveling the composition of POs from plastics. The additional values of GC×GC separations in classical petrochemical applications have been reported and proven in several works [50–54].

The first GC×GC use for the analysis of POs from plastics, in the authors’ knowledge, was reported in 2014 [48]. In this work, the main polymers of the initial plastic solid waste were PE, PP, PS, and polyamide (PA), containing trace amounts of food residuals as well. The authors exploited several detectors to achieve an unprecedented insight into the composition of plastics waste PO. MS was used for tentative identification purposes and selective detectors were exploited for the quantitative analysis. On the one hand, the use of a sulfur chemiluminescence detector (SCD) and a nitrogen chemiluminescence detector (NCD) allowed the accurate determination of S-containing and N-containing organic compounds. On the other, the use of an FID allowed for a more accurate quantitative determination of different hydrocarbons at the class level. The authors also compared the results obtained by GC×GC with the sample elemental composition obtained from elemental analyzers (see Table 1). The results among the two approaches were in very good agreement (except for the oxygen-containing compounds), with the GC×GC data having the advantage of providing detailed molecular information of single components.

**Table 1:** Elemental composition of plastic waste pyrolysis obtained with a conventional total organic compound (TOC) analyzer and GC×GC. Table from [48]

	Plastic waste pyrolysis oil (EA)	Plastic waste pyrolysis oil (GC×GC)
C (wt%)	88.4 ± 0.3	88.0
H (wt%)	9.6 ± 0.2	10.9
S (wt%)	0.17 ± 0.003	0.17^a^ ± 0.005
N (wt%)	1.08 ± 0.07	1.06^b^ ± 0.05
O (wt%)	0.99 ± 0.09	0.35

^a^Of which 0.14 wt% identified

^b^Of which 0.84 wt% identified

**Fig. 6:**
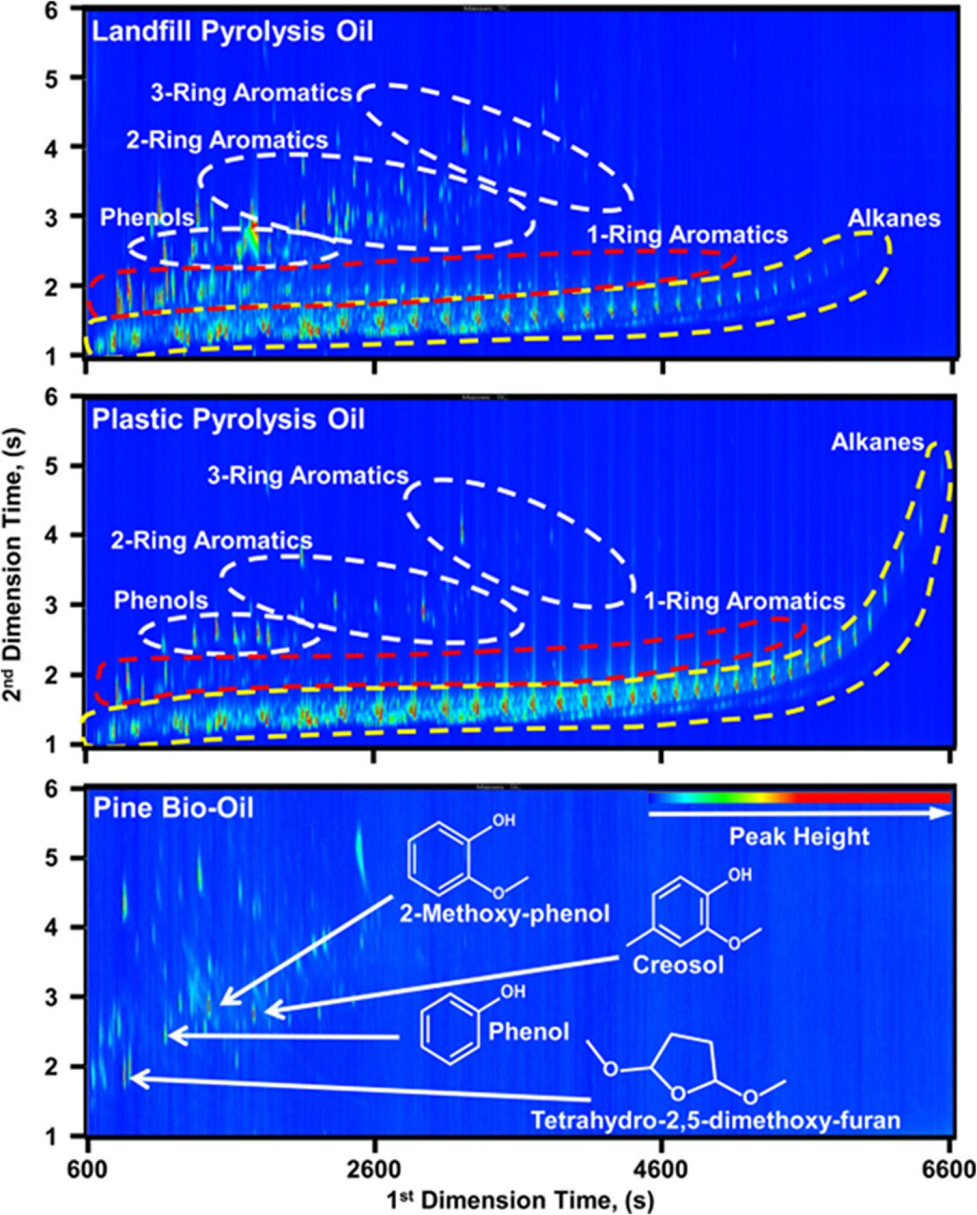
2D plots obtained from the analysis of landfill (top), plastic (middle), and pine (bottom) POs. The marks and dotted area highlight some of the constituents and chemical classes. Reproduced with permission from [53]

In 2017, three POs produced from landfill waste, recycled plastics, and pine forestry residue were compared, highlighting their different hydrocarbon composition [53]. Here, the authors combined elemental analysis, GC×GC–MS, FT-IR, FT ion cyclotron resonance MS (FT-ICR MS), and liquid chromatography for the comparison of samples. As can be observed from the GC×GC chromatograms in Fig. 6, there is a different hydrocarbon composition between plant- (pine) and non-plant-based (landfill and plastic) POs. Landfill and plastic POs show a range of alkanes and 1-ring aromatics similar to that of petroleum, as opposed to the pine-derived PO which was characterized by O-containing compounds (phenol, furan, and carbonyl functionalities) as degradation products of lignin, cellulose, and hemicellulose.

Among the landfill and plastic PO, the higher content of aromatics and phenols was probably related to the miscellaneous composition of landfill waste (29% combustibles, 23% paper, 13% plastic, and 11% wood). Recycled plastics were composed of PE, PP, and PS. Again, the molecular composition given by GC×GC plots has permitted to point out bidimensional patterns, which are distinctive sample fingerprints.

Interesting examples of quality evaluation of post-consumer plastics waste and its PO were also studied in other research studies [15, 54]. In this context, for PO quality, it is intended its suitability to be used as feedstock in the steam cracker for further production of plastic products.

**Fig. 7:**
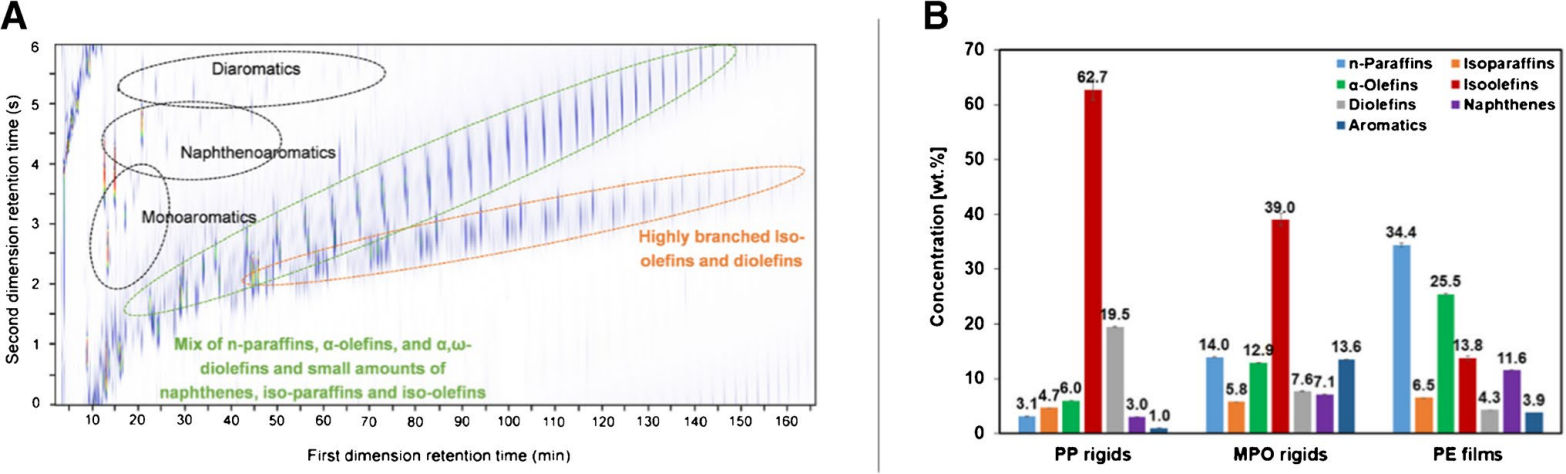
**A** GC×GC-FID chromatogram of MPO rigids PO; **B** PIONA analysis of the PO using GC×GC-FID. Reproduced with permission from [15]

Post-consumer plastics packaging waste fractions, namely mixed polyolefins (MPOs) rigids, PE films, and PP rigids, were processed in a continuous pilot-scale pyrolysis unit and POs subsequently characterized using GC×GC with multiple detectors (MS, FID, SCD, NCD, atomic emission detector AED) [15]. In this research, GC×GC-FID was used to quantify the entire hydrocarbon spectrum of respective POs. More than 1500 compounds were detected, and each group of compounds had a unique distribution pattern which helped to identify the individual compound groups. The authors highlighted the difficulty to perform these analyses in 1D GC, since most compounds would largely coelute, making the identification/quantification of minor compound groups, such as aromatics or naphthenes, impossible. The difficulty to identify minor compound groups by means of 1D GC is evident by considering that several other previous papers [32, 55] did not report naphthenes in plastic waste POs. For this very complex mixture with several isomeric structures, the use of less sophisticated separation techniques leads to an unresolved hump of compounds, e.g., saturated and unsaturated hydrocarbons, and therefore to inaccuracies in the analytical result [55]. The fact that diolefins as well as naphthenes are important drivers for coke formation in steam cracking justifies the importance of using the most advanced analytical techniques. A representative 2D contour plot is shown in Fig. 7A: such separation was obtained using a non-polar/polar column set (equivalent to 5% diphenyl/95% dimethyl polysiloxane and 35% diphenyl/65% dimethyl polysiloxane as primary and secondary column, respectively) and exploiting a very slow temperature program at 2 °C/min to increase the overall separation at the expense of analysis time (180’).

The composition of the POs differs significantly depending on the pyrolyzed plastic waste materials, as already indicated by the GC×GC-FID chromatograms. As can be seen from Fig. 7B, high concentrations of highly branched iso-olefins and diolefins indicate high PP concentrations in the original plastics waste material while high concentrations of linear paraffins and olefins indicate PE-rich waste. This can be explained by the different degrees of side groups in the polymer chains of PE and PP. The MPOs depict well the intermediate composition (about 50/50 PE/PP mixture), while the higher aromatic can derive from feedstock impurities such as PET or PS. The higher abundance of naphthenes in PE is instead explained by the higher probability of linear olefins to undergo Diels–Alder reactions, compared to branched olefins forming in PP.

Based on this detailed oil composition (wt%) and knowing that for subsequent steam cracking minimal amount of olefins, naphthenes, and aromatics are ideally sought, the authors also proposed the necessary dilution ratios with other pure feedstocks for the upgrading of the different POs under study: the PE films, thanks to the lower olefins amount, required less dilution for further upgrade and use.

Regarding the concentration of heteroatoms such as nitrogen, sulfur, oxygen, and halogens, strict specifications are in place for steam-cracking feedstocks. This has been more historically studied and established for fossil-based feedstocks, but it is accepted for plastic waste POs as well [16].

These impurities are indeed causing corrosion, fouling, and increased coke formation, as well as downstream catalyst deterioration.

In the research studies previously described [15, 54], detailed elemental compositions were derived exploiting selective detectors hyphenated with GC×GC. Considering the three PE, PP, and MPO feedstock POs, the high amounts of heteroatom-containing compounds indicate that current sorting and recycling lines, including washing, are not able to remove such elemental contaminants completely. For example, residual paper and biomass contamination, as well as additives, potentially contribute to heteroatom and metal content. N, S, O, and Cl values measured in the plastic waste feedstocks further indicate probable contamination of feedstocks by heteroatom-containing polymers such as PET, polyamide (PA), polyurethane (PUR), and polyvinyl(idene) chloride (PV(D)C).

As mentioned before, the high-resolution and multi-dimensional chromatographic process is a crucial step for the separation of single isomers in these complex hydrocarbon mixtures. On the other side, to allow the characterization of these hydrocarbon mixtures, information on the chemical structure must be retrieved using mass spectrometry.

**Fig. 8:**
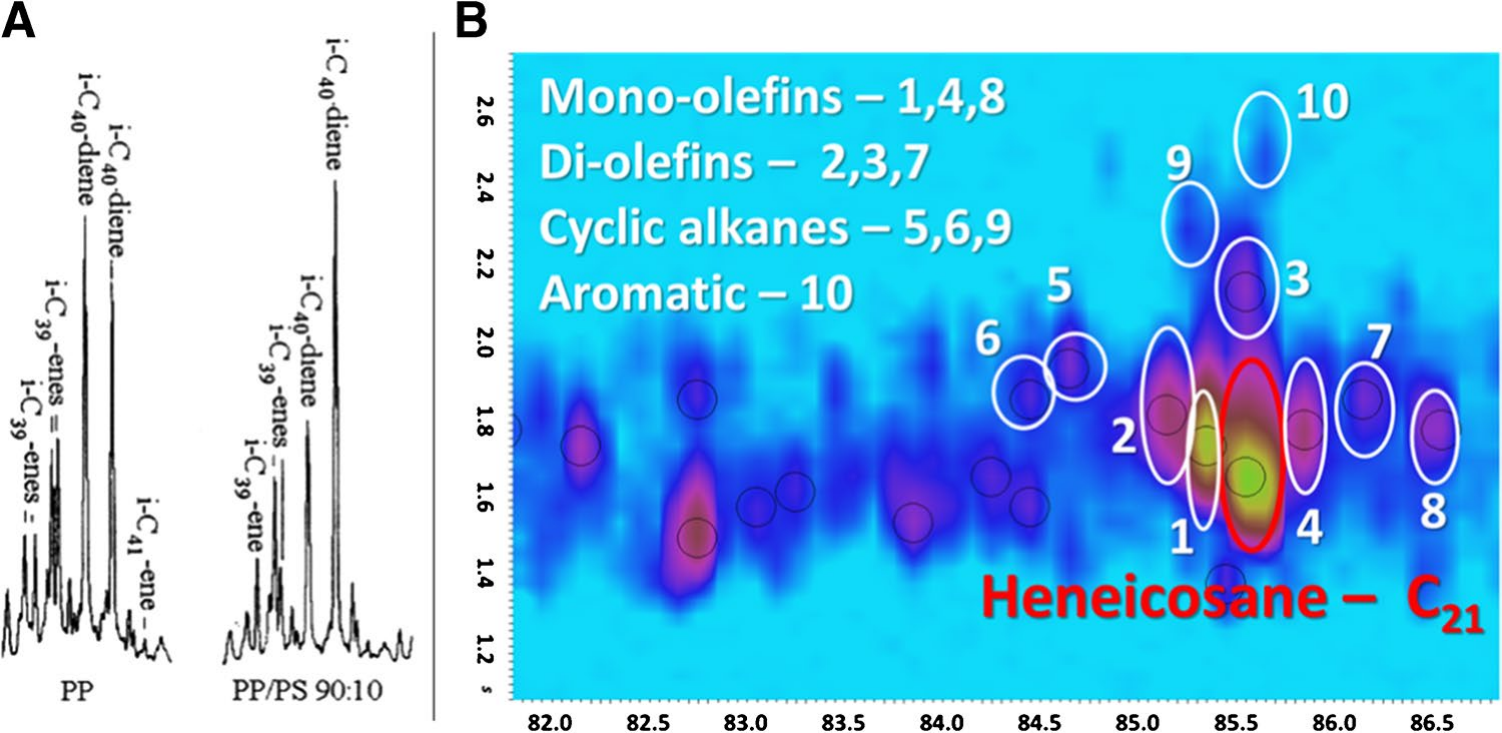
**A** GC–MS chromatogram expansion of the C_40_ group from the pyrolysis of a PP and PP/PS (reproduced with permission from [51]); **B** GC×GC-PI-HRTOFMS chromatogram expansion of the C_21_ group (adapted from [58])

Despite the use of high-resolution MS, the pre-separation of analytes is very important to obtain pure spectra for their tentative identification [56]. Even when the separation is successful, compound identification can be challenging. In these cases, an important aid can be given by milder ionization conditions [57]. A recent study reported the use of soft photo-ionization (PI) HR MS coupled to GC×GC for deep insights into the isomeric composition of POs from the pyrolysis of mixed municipal plastic wastes [58]. In this, the authors exploited a 2D separation using a non-polar/polar column set (equivalent to 5% diphenyl/95% dimethyl polysiloxane and 35% diphenyl/65% dimethyl polysiloxane as the primary and secondary columns, respectively). The fragmentation of various aliphatic (saturated, unsaturated, cyclic, and branched) and aromatic hydrocarbons was thoroughly investigated and compared between PI and EI. Figure 8 reports in detail and side-by-side typical clusters of isomers around the principal linear paraffin (C_40_ and C_21_), in conventional GC and GC×GC separations, respectively. Clearly, the separation in the second dimension allows for a more detailed distinction of species (Fig. 8B), which otherwise would be hidden and would appear as a hump or lump, as illustrated in a conventional 1D GC separation in Fig. 8A.

An advanced application involving GC×GC was reported recently, exploiting the use of a two-stage reactor coupled online ahead of the chromatographic system to study the thermal decomposition or catalytic cracking of conventional and renewable feedstocks [59]. With this special configuration, the authors evaluated the performance of industrial formulations of steam-treated fluidized catalytic cracking (FCC) catalysts and zeolite-based (HZSM-5) catalyst additives for the in-line catalytic upgrading of polyolefin pyrolysis products towards light olefins and aromatics in two steps. Virgin LDPE and MPO waste were used as feed materials for pyrolysis. The reactor apparatus consisted of a micropyrolyzer from which vapors were swept by the carrier gas over a second chamber containing the catalyst (the upgrading reactor), and subsequently entering the GC, in which the analytes were focused using a cryotrap. A splitter allowed the analysis of light fractions (C_2_–C_4_) into a separate multi-column GC for light gas analysis, while the heavier fraction was evaluated into the GC×GC.

This special configuration allowed both the analysis of light fractions (C_2_–C_4_) diverting the flow to a separate multi-column GC for light gas analysis and of heavier fractions.

This is an example of an analytical scale “laboratory-based” experiment which can be scaled up. Otherwise, examples of the use of chemical composition to monitor and tailor processes in pilot plants exist, like process GC. The results reported in this article are, on the one hand, highly relevant for the design of plastic waste pre-treatment steps prior to thermochemical recycling. On the other hand, they are important for the design of upgrading steps of the POs to produce suitable feedstocks for thermocatalytic recycling pathways such as steam cracking.

Another increasing field of application, where in our opinion GC×GC will greatly contribute, is the safety assessment of “novel” recycled products. Indeed, as more types of plastics are recycled, there will need to be new ways to ensure that additives in these materials are not introduced into consumer products (fragrances, flame retardants, solvents, biocides, dyes, etc.) [60, 61].

**Fig. 9:**
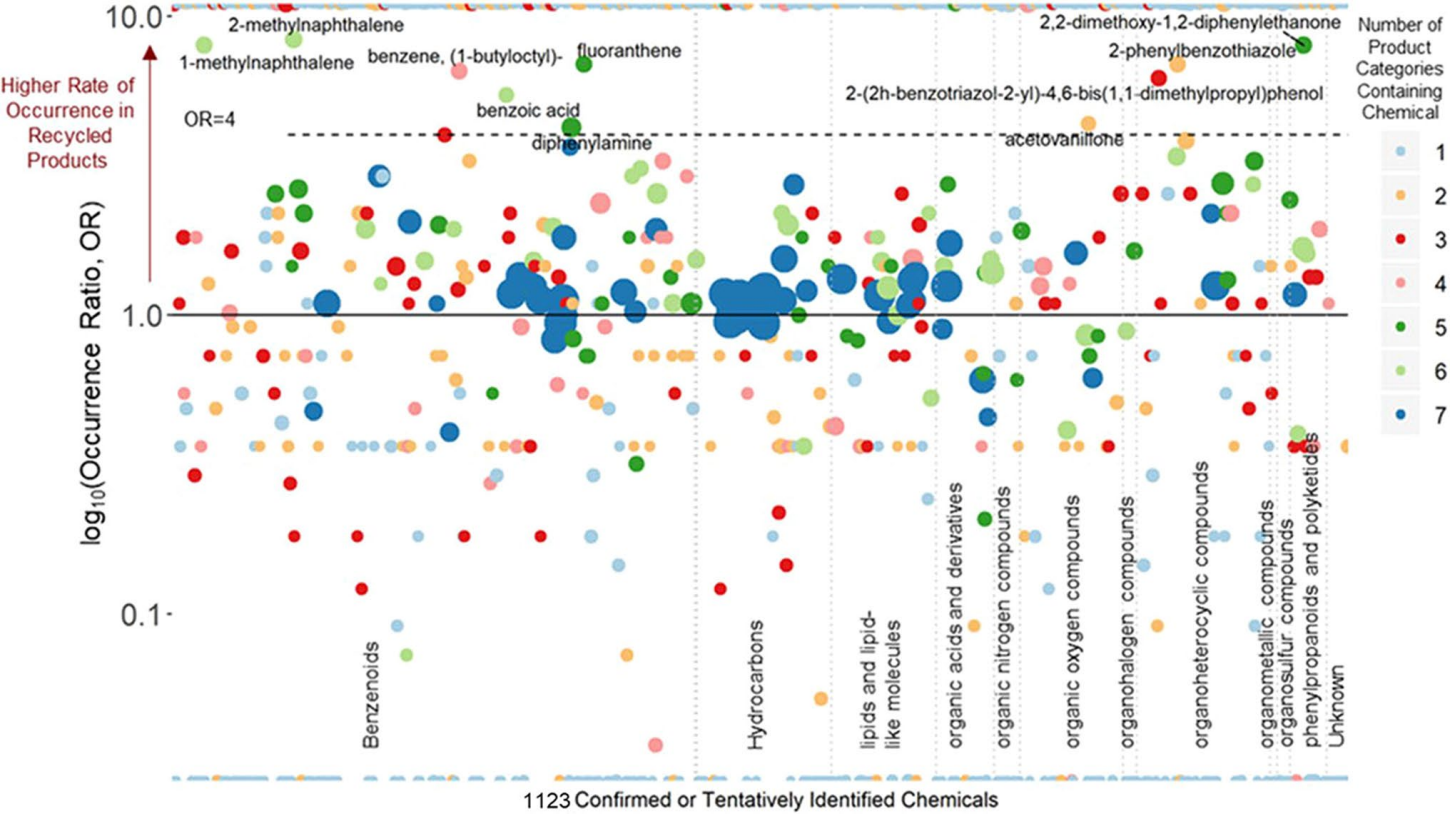
Occurrence ratios (OR) of 1123 chemicals measured in virgin and recycled products. OR is defined as the fraction of recycled products with occurrence divided by the fraction of virgin products with occurrence. The size of the points indicates the number of individual products with occurrence; the color indicates the number of product categories overall with at least one occurrence. Chemicals with a high occurrence ratio (> 4) are labeled. Points at the very top and bottom of the graph are chemicals with an OR of infinity (no occurrence in virgin products) and 0 (no occurrence in recycled products), respectively; these chemicals occurred in relatively few products and categories. Reproduced with permission from [60]

In a recent report, Lowe et al. [60] performed a suspect screening analysis to characterize the composition of more than 200 household items (both from recycled and virgin materials), falling into seven categories: plastic children’s toys (e.g., bath toys, teethers) or play mats; paper products (e.g., construction paper, copy paper); fabric-containing clothing and home goods (e.g., t-shirts, rugs); plastic food contact materials (e.g., boards, sandwich bags); non-culinary use plastic household items (e.g., hangers, dog bowls); construction materials, including vinyl flooring, lumber, and boat-board (a thick plastic sheeting used in marine and vehicle applications); and residential products manufactured using recycled tire-derived material (e.g., turf mats, rubber mulches) [60]. The potential main chemicals and their occurrence in recycled products were highlighted, as pictorially summarized in Fig. 9. Phthalate plasticizers were detected in both virgin and recycled products. Di(2-ethylhexyl) phthalate (which has been proposed as an indicator of phthalate contamination in recycled plastic) was found with more occurrence in recycled plastic products (7:1 ratio). Three brominated flame retardants were detected in a black recycled plastic clothing hanger. Other chemicals, such as 2-ethylhexyl diphenyl phosphate, triphenyl phosphate, and tris(2-chloroethyl) phosphate, were detected in 37 recycled products, including food contact materials, children’s products, construction materials, and plastic home/auto products. Thirteen PAHs were detected in recycled tire products, as well as five additional PAHs. It must be said that, even though the results of this study showed the presence of several potentially hazardous chemicals in recycled products, a potential exposure assessment is not possible without information regarding leaching or migration from the products.

## Final remarks and outlook

More and more demanding studies are arising in the plastics recycling field, representing a major challenge for conventional separation methods. The proper use of hyphenated and high-resolution separations (particularly, GC×GC–MS) can undoubtedly unravel the complexity of those recycled fluid streams (or pyrolysis oils) for their optimal use as feedstocks for the generation of new goods, thus supporting the quest for circular economy in closing the loop of plastics. This is a dynamic and evolving issue because of the variability of the starting plastics to recycle, needing the most advanced and versatile analytical techniques.

A truly comprehensive analysis, even though in this case is restricted to only the GC-amenable analytes (volatile and semi-volatile organic compounds), could encompass many types of testing, or as subsets. For example, both the detailed hydrocarbon analysis (e.g., paraffins, olefins, cyclics, aromatics) and the contaminant/impurity determination (e.g., heteroatom- and halogen-containing compounds) can be obtained in a single analysis, with the possibility of relieving R&D and QA/QC laboratories from numerous chemical investigations.

The detailed composition of the downstream intermediate, such as pyrolysis oils, is important for the design and optimization of the process itself. There is a tremendous potential to advance the existing chemical recycling processes and to design new ones by using the information from the detailed characterization of pyrolyzates and related kinetic modeling. The R&D laboratories expanding or starting their GC×GC capability will certainly secure advanced knowledge for the development, control, and process optimization for the revalorization and transformation of plastic waste into new products.

The remarkable development of artificial intelligence and machine learning to handle the high-density and detailed chemical data generated will probably open new correlations with physical properties (similar to simulated distillation). To this end, the implementation of detailed kinetic models for simulation and process optimization (e.g., thermocatalytic cracking for chemical recycling), containing much more molecular chemical details, is expected in the future.

Almost 32 years from its inception, GC×GC represents a mature and reliable analytical tool to handle the complexity of POs from plastics thanks to the dedicated solutions and the stability of hardware and software from a variety of providers. GC×GC is already present in the R&D laboratories of the most important players in the field (e.g., traditional fossil-fuel-based facilities turning into modern petrochemical industries focused on plastic-source feedstocks), and it is expected to expand further into other research centers and to penetrate even more into routine laboratories for quality control purposes. A limitation is still the lack of qualified personnel and the conservative/proprietary nature of the industry, which make slow this transition.

Finally, GC×GC coupled to mass spectrometry will play a central role in screening and analysis for contaminants and non-targeted constituents in recycled materials and derived products.
